# Estimating emergency department crowding with stochastic population models

**DOI:** 10.1371/journal.pone.0295130

**Published:** 2023-12-01

**Authors:** Gil Parnass, Osnat Levtzion-Korach, Renana Peres, Michael Assaf

**Affiliations:** 1 Racah Institute of Physics, Hebrew University of Jerusalem, Jerusalem, Israel; 2 Headquarters, Shamir Medical Center, Be’er Ya’akov, Israel; 3 The Hebrew University Business school, Hebrew University of Jerusalem, Jerusalem, Israel; University of Hong Kong, HONG KONG

## Abstract

Environments such as shopping malls, airports, or hospital emergency-departments often experience crowding, with many people simultaneously requesting service. Crowding highly fluctuates, with sudden overcrowding "spikes". Past research has either focused on average behavior, used context-specific models with a large number of parameters, or machine-learning models that are hard to interpret. Here we show that a stochastic population model, previously applied to a broad range of natural phenomena, can aptly describe hospital emergency-department crowding. We test the model using data from five-year minute-by-minute emergency-department records. The model provides reliable forecasting of the crowding distribution. Overcrowding is highly sensitive to the patient arrival-flux and length-of-stay: a 10% increase in arrivals triples the probability of overcrowding events. Expediting patient exit-rate to shorten the typical length-of-stay by just 20 minutes (8.5%) cuts the probability of severe overcrowding events by 50%. Such forecasting is critical in prevention and mitigation of breakdown events. Our results demonstrate that despite its high volatility, crowding follows a dynamic behavior common to many systems in nature.

## Introduction

We live in a crowded world. Crowded environments such as shopping malls, trains during rush hours, airports, performance venues, religious sites, and hospital-emergency-departments [1–3] are characterized by an influx of arriving individuals, each seeking to receive a service that is often customized to their needs, and sometimes includes clear prioritization criteria. As a result, the exit rate, and thus the number of individuals present at any given moment, greatly fluctuates across hours and days, with high volatility and sudden "spikes" of overcrowding.

Modeling crowding has been a major challenge in disciplines such as operations research, marketing, transportation research, psychology, and healthcare management [4–21].

A considerable body of research focused on describing the average behavior in the population (e.g., arrival rate, length of stay, probability of making a specific decision based on crowding etc.). These models typically implemented methodologies such as queuing models [11,12], econometric analysis [4–9], time series analysis [10] (Kadri, Harrou, Chaabane, & Tahon, 2014), dynamic choice models [13,14], and performance analysis [16] (Chan, Farias, Bambos, & Escobar, 2012). While these methods are powerful for capturing average behaviors, they are less suitable for analyzing the fluctuating nature of crowding. Indeed, as demographic fluctuations typically scale as the square root of the population size, such models become less accurate in modeling environments with smaller populations. In particular, these models do not account for outliers, bursts, and large overcrowding spikes, which characterize crowding dynamics, and at their extreme may lead to catastrophic events such as unreasonable waiting times, service breakdown, or crowd disasters.

To better capture the stochastic nature of crowding, a class of discrete event simulation studies [18,22–26] has been proposed. These models typically simulate a specific context (e.g., transportation choices by passengers [23], hospital-emergency-department [18,22,26], and pedestrian crowds congestion [24]), by decomposing it into stages, defining the inter-stage transition flow, and examining this flow subject to the parameters of the environment. For example, Kuo et al 2016 develop a simulation model in order to improve the efficiency of a Hong Kong hospital ED [18]. The simulation mimics the journey of a patient through the various stages of the ED process, incorporating a large number of ED parameters, and inferring service-time distributions from the data. Such models are powerful and allow exploration of ED operations aspects; yet, due to the large number of context specific parameters they use, their output is valuable only when the model closely resembles the real system [27].

Other methods which incorporate stochasticity are based on machine-learning algorithms [15,19,28,29]. While these methods provide better predictions, their “black box” nature makes it harder to draw interpretable insights. Moreover, lacking an analytical framework, these models provide less insight into the relative effect of each of the model’s parameters on overall crowding [19].

Here we suggest a different approach to modeling crowded environments. We show that despite its high volatility and spiky nature, crowding can be accurately described using a simple, generalizable model, with a minimal number of parameters. Our model formulates a Langevin stochastic differential equation [30,31], which describes the dynamics of the number of patients at any given moment, and includes noise terms which account for heterogeneity in the incoming individuals, as well as variability from the side of the service provider. While the model is compact and its basic version can be solved analytically, it captures both the average behavior as well as the stochastic nature of crowded environments, including large deviations and spikes, while still enabling conducting parameter exploration and gaining key insights on the formation of crowding and the possible avenues for its mitigation.

Rooted in population dynamics, our approach connects individual-level responses with changes in population density and structure [32], as well as environmental variability. These models have been used to describe fluctuating population dynamics in ecology [33,34], population biology [35,36], epidemiology [37,38], cell biology [39–44], statistical physics [45], and even turbulence [46–48]. This paper is a novel attempt to use them to address crowding effects.

We implement the model in the context of hospital emergency departments (EDs). ED crowding constitutes a prevalent, enduring, and progressively escalating global challenge worldwide [49]. EDs regularly face crowding [50–52], with many experiencing daily occurrences of overcrowding conditions [53]. ED overcrowding has been shown to harm patient satisfaction [54,55], and lead to compromised safety, timeliness and effectiveness of patient care [4,5,56–58], patient attrition [6,56,59], and even higher mortality rates [9,56]. ED crowding is a major factor in the fatigue of medical staff [50], and a contributing factor to violent incidents [60]. Therefore, mitigating ED crowding has been a top priority for health authorities and policy makers [56,61].

ED crowding has been intensively studied [10–13,15,16,18–22,28,29], exploring a variety of operational metrics [27], and using variety of modeling techniques (see Hu, Barnes and Golden 2018 [20], Vanbrabant et al. 2019 [27], and Sinreich and Marmor 2005 [25] for review). Our model offers a fourfold contribution: First, it captures average behavior, as well as spikes and events of overcrowding, which received less research attention [10–13]. Second, our model is compact. Compared with the discrete event models [18,25], it uses only two parameters, and can offer a closed-form solution and therefore a more generalizable outlook. Third, being based on a general Langevin formulation, it allows comparison with a broad class of population-related phenomena in other research domains. Four–unlike machine learning models [15,19,28,29], our model is interpretable and enables to test the impact of changes in specific variables on the overall outcomes. Therefore, it complements the literature to provide a more generalizable and interpretable understanding of crowding, explore spikes and outliers, and connect crowding population behaviors.

We test our model on a proprietary dataset which includes the complete set of records of 679,762 ED visits over five years. We find that despite its high volatility and spiky nature, ED crowding follows a dynamic behavior common to many systems in nature. The model provides reliable forecasting of the average as well as the overall hourly crowding distribution. More importantly, due to its analytical nature, the model can predict how the overcrowding probabilities vary with the model parameters. This ability, absent from various discrete event and machine learning models, is an important tool in understanding ED overcrowding and is a first step towards exploration of means for prevention of ED breakdowns.

## A stochastic population model for crowding

We first present the basic notations and dynamics through a mean-field deterministic model, wherein noise is ignored, and then develop the complete stochastic model. The mean-field deterministic representation is formally valid in the limit of an infinite population.

### Mean-field deterministic model

Assume a service venue where individuals arrive at flux rate denoted by *f*(*t*) and exit at rate *β*(*t*). The dynamics of the mean number of individuals $\bar{n}(t)$ in the venue reads:

$$\frac{d\bar{n}(t)}{dt}=f(t)-\beta (t)\bar{n}(t).$$ (1)

Here, both the arrival flux and exit rate explicitly depend on time, as these constantly vary during the day. Starting with *n*_0_ individuals, the solution to Eq (1) reads:

$$\bar{n}(t)=e^{-\int_{0}^{t}\beta (s)ds}\int_{0}^{t}f(s)e^{\int_{0}^{s}\beta (r)dr}ds+n_{0}e^{-\int_{0}^{t}\beta (s)ds}$$ (2)

Solution (2) radically simplifies by approximating *f*(*t*) and *β*(*t*) by their time-averages: $f(t)=\bar{f}$, and $\beta (t)=\bar{\beta }$. In this case, $\bar{n}(t)=n_{0}e^{-\bar{\beta }t}+n_{*}(1-e^{-\bar{\beta }t})$, i.e., the mean number of individuals converges, after a timescale of $O(\beta^{-1})$, to the stable fixed point at $n_{*}=\bar{f}/\bar{\beta }.$

### Stochastic model

In actual crowded environments, besides their deterministic variations, the arrival flux and exit rates contain a stochastic component. We therefore incorporate two types of noise into the mean-field dynamics: inter-individual, and systematic. The first type, inter-individual noise, emanates from heterogeneity in the arrival flux, discreteness of individuals, or the type of service sought by each individual, and is sometimes termed *demographic*, or *internal*. To account for the latter, we write down the so-called *master equation*–a gain-loss equation describing the evolution of the probability *P*_*n*_(*t*) of observing *n* patients at time *t*, where time is continuous. For the process which includes influx and exits at rates *f*(*t*) and *β*(*t*) respectively, the master equation reads [31]:

$$\frac{dP_{n}(t)}{dt}=f(t)[P_{n-1}(t)-P_{n}(t)]+\beta (t)[(n+1)P_{n+1}(t)-nP_{n}(t)].$$ (3)

A useful approximation of the master equation, valid in the limit of large *n*, is the so-called *Langevin equation* [31] [see SI, Appendix A in S1 File]–a stochastic differential equation for the momentary number of patients at the venue. For the stochastic process at hand, the Langevin equation reads [31]: $dn(t)/dt=f(t)-\beta (t)n(t)+\sqrt{f(t)+\beta (t)n(t)}\xi_{1}(t)$. This equation includes a deterministic term, *f*(*t*)−*β*(*t*)*n*(*t*), identical to Eq (1), and a noise term, $\sqrt{f(t)+\beta (t)n(t)}\xi_{1}(t)$, representing the inter-individual noise [30,45] [see SI, Appendix A in S1 File], where *ξ*_1_(*t*) is a stochastic variable defined below.

Notably, our model also includes a second noise source which is absent from the above Langevin equation. This noise, termed *systematic noise* or *external noise*, is caused by changes in the facility infrastructures, variations in the number and quality of staff, and inconsistencies in organizational procedures. With this additional noise term, which directly affects the exit rate from the ED, the Langevin equation becomes:

$$\frac{dn(t)}{dt}=f(t)+\sqrt{f(t)+\beta (t)n(t)}\xi_{1}(t)-\beta (t)[1+\xi_{2}(t)]n(t),$$ (4)

where apart from the deterministic and demographic-noise terms, we added to the model a second noise term, *β*(*t*)*ξ*_2_(*t*)*n*(*t*) which corresponds to the systematic noise. It is multiplicative and scales with the population size [62], as it equally influences all individuals. We assume that the two stochastic variables, *ξ*_1_(*t*) and *ξ*_2_(*t*), are mutually independent, zero-mean, delta-correlated (in time) noise terms with magnitudes *σ*_1_ and *σ*_2_, respectively, such that 〈*ξ*_*i*_(*t*)〉 = 0, and $\langle \xi_{i}(t)\xi_{i}(t+\tau )\rangle =\sigma_{i}^{2}\delta (\tau )$, where *δ*(*τ*) is the Dirac delta function. Notably, we have confirmed that taking *ξ*_*i*_ with finite correlation time (i.e., colored noise, when *ξ*_*i*_ satisfies, e.g., an Ornstein-Uhlenbeck equation [31]), does not qualitatively change the model’s results [see SI, Appendix B in S1 File]. Here, it is important to note that, a qualitatively similar equation to Eq (3) can in principal be obtained using methods from queuing theory, by taking a processor-sharing server with capacity scaling linearly with the population size *n*, and adding external noise to the capacity [63].

The solution of Eq (4) provides the time trajectory of the momentary number of patients, *n(t)*, for a single noise realization. However, since we are interested in the complete statistics of events, including events of extreme overcrowding, we solve the equation for multiple realizations. A histogram over the different realizations provides the complete statistics of events. Alternatively, this histogram can be computed by transforming Eq (4) into a Fokker-Planck equation [31] (see SI, Appendix A in S1 File).

## ED data

We assembled a unique dataset containing the complete records of 679,762 visits by 333,471 unique patients, which are all the ED visits between January 1st, 2013 and June 30, 2018, at the Shamir Medical Center, a large state-run Israeli hospital. Each record represents a visit by a single individual, and for each such record the dataset contains, inter alia, an encrypted patient identifier (to enable tracking of revisits), reason for visit, gender, age, mode of arrival (ambulance vs. self-arrivals), triage urgency, lab test timings and results, and all of the medical decisions for the patient. The data include time of arrival to and time of departure from the ED (discharge or hospitalization), as well as time stamps for each recorded operation; thus, each visit-log contains a minute-by-minute description of the patient visit. 46.4% of the patients were females, 24% under age 18 and 23% age 65 or older. The most common reasons for arrival are sickness (72% of cases) and injury (16%). The average length of stay in the ED is 3.9 hours, with a standard deviation (STD) of 2.6 hours.

**Fig 1:**
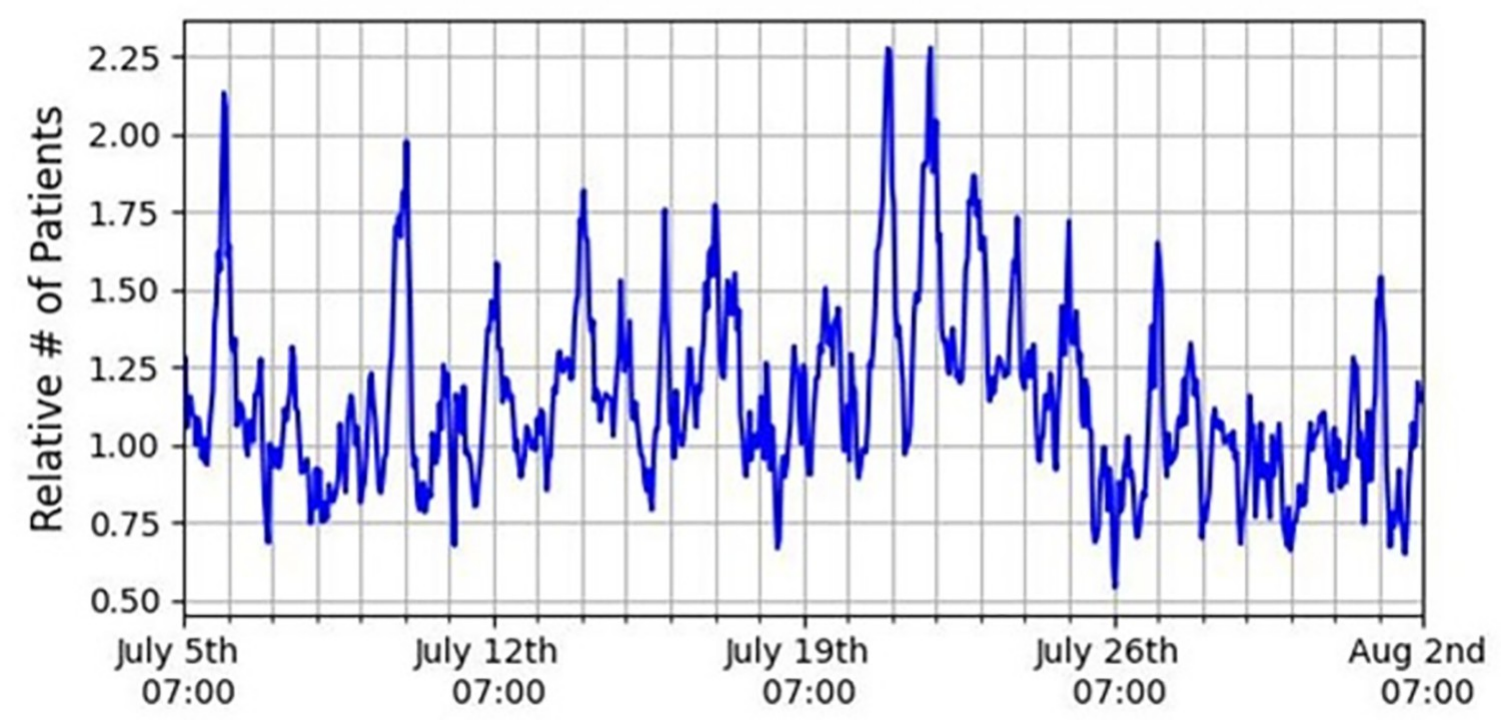
Relative number of patients (number of patients divided by their hourly mean) as function of time for a typical month of the data (July 5th until August 2nd, 2015).

We use the weekly hour (e.g., Sunday 8:00–9:00) as a basic time unit, due to the population’s strong weekly cycle, with high typical crowding during weekdays (Sunday-Thursday, following the Israeli workweek), and lower crowding on the weekend (Friday-Saturday). To align with the ED shift structure, we grouped the hours, when needed, into morning (7:00–15:00), afternoon (15:00–23:00), and night (23:00–7:00) shifts. To demonstrate the spiky nature of the data, we show in **Fig 1** the momentary number of patients normalized by their hourly mean, for a typical period of 4 weeks. During this period, one has ~15−16 spikes exceeding the mean by 50%, and ~4 spikes exceeding the mean by 100%, indicating a quite significant noise level.

## Crowding metrics

Research and practice suggest various ways to measure ED crowding, differing in the data they require and purpose of measurement. Some methods capture the inflow of patients (total number of daily visitors, current number of patients being treated or waiting to be seen), while others capture the load experienced by the patients or the staff (waiting times, treatment times, patients who leave without being seen, nurses being rushed or feeling rushed, etc.). A considerable number of measures focus on the facility’s physical infrastructures (number of available beds, capacity in observation area, patients placed in ED hallways, etc.), see [64] for review.

A popular measure is the National Emergency Department Overcrowding Score (NEDOCS) [61], officially used by the USA federal authorities. This score is a multi-variable function based on both site-specific parameters (total beds in the ED, Number of hospital beds), and momentary indices (total number of patients, average waiting time, etc.). For this research, we measure four metrics: arrival flux, exit flux, momentary number of patients, and ’’patient hours’’.

**Fig 2:**
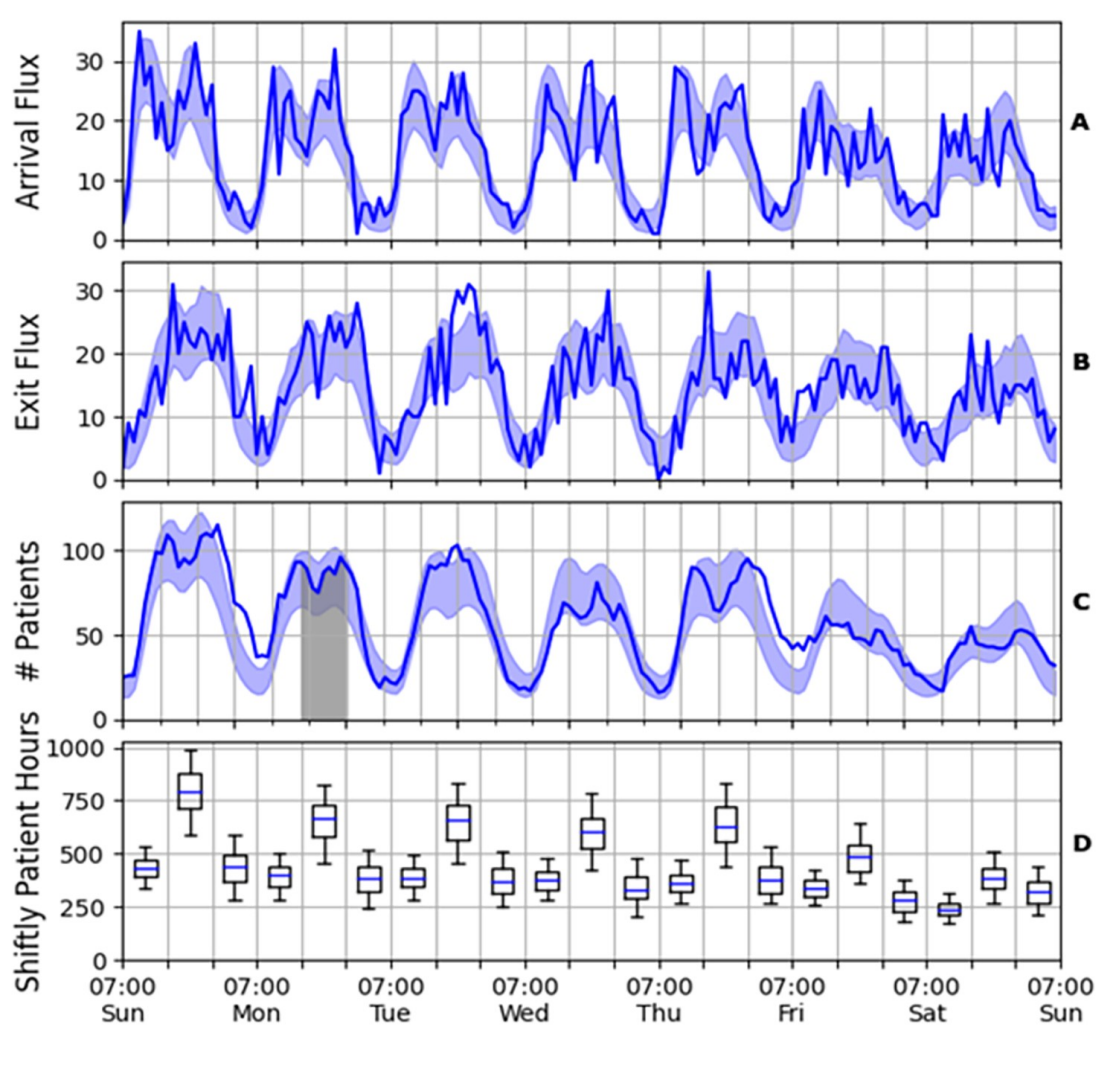
Timeline of the arrival flux (A), exit flux (B), and momentary number of patients in the ED (C). The solid line is the hourly value for a typical week (July 5–12, 2015); the shaded blue area denotes one standard deviation from the average over all weeks. The grey area in (C) denotes the value of the patient hours for the Monday afternoon shift. (D) Distribution of patient hours per shift. The box extends from the 25th to the 75th percentile of the shift data (whiskers mark the 5th and 95th percentile), with a line at the median. A week begins at 7:00 on Sunday, while the vertical grid–lines represent the shifts (07:00, 15:00 and 23:00).

The arrival flux *f*(*t*) is the hourly number of incoming patients (**Fig 2**A). The exit flux is the number of patients who left the ED per hour for both discharge and hospitalization, see (**Fig 2**B). The momentary number of patients is the number of patients who are currently in the ED, denoted by *n*(*t*) (**Fig 2**C). Finally, the metric of patient hours, which was specifically developed for this research, is defined by $C=\int_{T}^{T+\Delta T}n(t)dt$ (see the grey area under the curve in **Fig 2**C and **2**D). This is the accumulated number of patients in the ED during the time interval [*T*, *T*+Δ*T*], where T is measured in hours. That is, if the shift began with 100 patients, and they all remained throughout the shift, the patient hours for that shift will be 800. While the measure of patient hours, which is a combination of several other commonly used measures [65], does not account for factors such as bed availability, waiting times, etc., its key advantage is that it is easier to integrate over shifts and days, and therefore, is more suitable for forecasting future ED occupancy.

## Crowding statistics

The average hourly arrival flux of patients in our data is 14 (STD = 8, min = 0, max = 46). The average hourly exit flux of patients is likewise 14 (STD = 7, min = 0, max = 46). The fact that their average coincides naturally indicates that there is no long-term accumulation of patients. The average momentary number of patients present in the ED (as recorded hourly) in our data is 55 (STD = 27, min = 3, max = 161), while the average patient hours per shift is 440 (STD = 164, min = 130, max = 1,198).

**Fig 2** demonstrates strong daily and weekly cycles. Most of the patients arrive in daytime, and most of them, even those who arrived in the afternoon or evening, tend to leave before the late nighttime. During weekends, there are fewer patients in the ED than during the week. **Fig 2**D depicts the distribution of patient hours per shift. The afternoon shift (15:00–23:00) is typically more crowded than the morning or night shifts, and the workweek is more crowded than the weekend, with the first day of the workweek (Sunday) being the most crowded.

**Fig 3:**
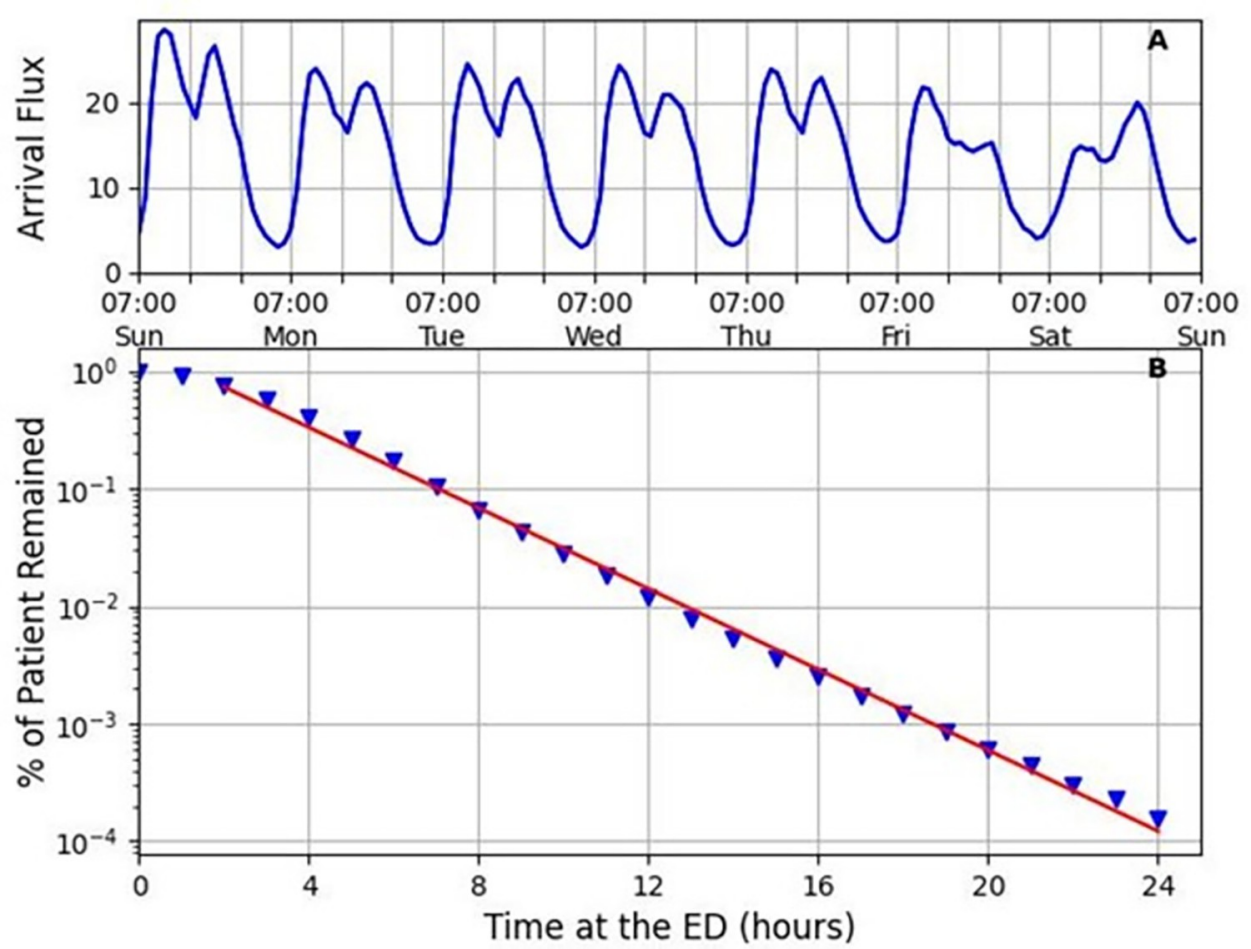
The functional shape of *f*(*t*) and *β*(*t*). (A) The average hourly arrival flux over a week. (B) The probability of a patient to remain in the ED since arrival versus time (a semi–logarithmic plot). The red line is an exponential approximation, indicating that the patients’ exit process is a Poisson process, with an expected value that equals the patients’ average length of stay.

**Fig 3** shows the average hourly arrival flux over a week and the probability of a patient to remain in the ED since arrival. The latter is illustrated in **Fig 3**B, where for each cohort of patients arriving at a given time *t*_0_, shown is the percentage of the remaining patients at *t*>*t*_0_. The graph exhibits a clear exponential-like decay with a constant decay factor *β*>0. It is convenient henceforth to define the exit flux and exit rate as the hourly number and hourly fraction of patients who left the ED, respectively. Thus, *β*(*t*) represents the exit rate, or the rate at which a patient exits the ED (discharged, hospitalized) within the next hour. The higher *β*(*t*) is, the faster the patient turnover, and therefore *β* may be a good metric for the ED efficiency.

## Estimating crowding

### Estimation procedure

The model in Eq (3) requires the estimation of the arrival flux *f*(*t*), the exit rate *β*(*t*), and the noise magnitude parameters *σ*_1_ and *σ*_2_. We take *f*(*t*) as the empirical arrival flux, averaged over the various weeks (**Fig 3**A). Notably, other theoretical choices of the arrival flux are possible, for example, by fitting the data to a trapezoid function of time for each day. We confirmed (see SI, Appendix C in S1 File) that this choice provides results with comparable accuracy to those shown below.

For the exit rate *β*(*t*), the data indicate that individuals exit the ED in a Poisson manner, i.e., the probability of not exiting until time *t* is given by *p*(*t*) = *e*^−*βt*^ (**Fig 3**B). That is, the individual exit rate can be regarded as a constant number *β* that depends on the specific day of the week (altogether four *β* parameters: Sunday, midweek (Monday-Thursday), Friday, and Saturday).

**Fig 4:**
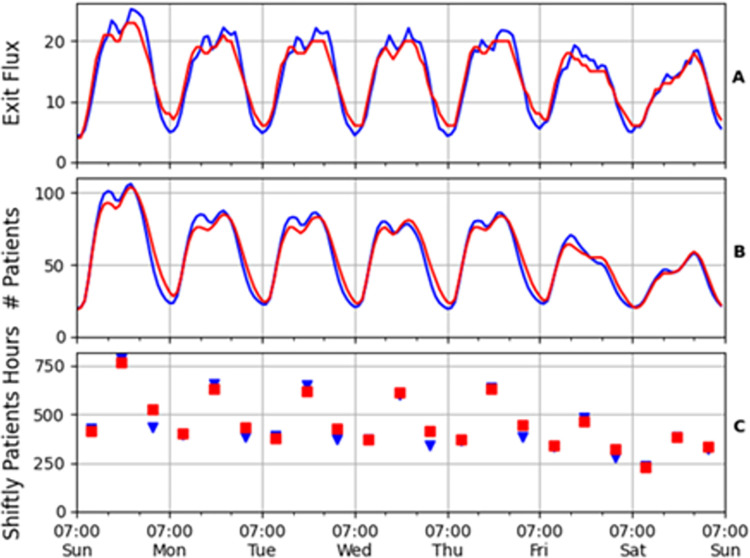
Comparison between the data (blue) and the model (red) for the average exit flux (A), average momentary number of patients (B) and the average patient hours per shift (C). Here *R*^2^ equals 0.929 (A) and 0.956 (B).

We estimated the model parameters using a two-step process: first, we estimated the deterministic *β* parameters, and then the stochastic components *σ*_1_, *σ*_2_. The *β* parameters were estimated using solution (2) to the mean field Eq (1) for the constant *β* case. The estimated *β* values are presented in **Table 1**. **Fig 4** shows the model’s results for the mean exit rate (**Fig 4**A), mean number of patients (**Fig 4**B) and mean patient hours (**Fig 4**C) compared with empirical data.

**Table 1:** Fitted parameter values for the model. The confidence interval represents the range of parameter values within 1 standard deviation (STD).

Stage I Deterministic	Parameter	Value	Confidence Interval (1 STD)
	*β* ^*Sun*^	0.224	0.003
	*β* ^M*id*^	0.244	0.002
	*β* ^*Fri*^	0.280	0.006
	*β* ^*Sat*^	0.304	0.009
Stage II Stochastic	*σ*_1_	1.1	0.015
	*σ*_2_	0.36	0.01

In the second step, we used the values of *β*, and applied maximum likelihood estimation to fit the noise parameters, *σ*_1_ and *σ*_2_. For every point in their two-dimensional parameter space ***σ*** = (*σ*_1_, *σ*_2_), we ran 10^4^ realizations of a simulated week, calculating the hour-by-hour STD, denoted by *S*(***σ***,*t*), and compared it to the data hourly STD, *S*_*i*_. Here, *S*_*i*_ = *S*_*i*_(*t*_*i*_) is comprised of *n* points of time denoted by *t*_*i*_ (measured in hours). We estimated ***σ*** by maximizing the Likelihood function:

$$L(\sigma ,p|{\left\{t_{i},S_{i}\right\}}_{i=1}^{n})={\left(2\pi \sigma^{2}\right)}^{-\frac{n}{2}}\prod_{i=1}^{n}\exp\left[-\frac{{\left(S_{i}-S(\sigma ,t_{i})\right)}^{2}}{2p^{2}}\right],$$ (5)

where we assumed that the sampled data have additional white Gaussian noise with variance *p*^2^. The Gaussian assumption is justified as the hourly arrival flux exhibits a Poisson distribution, which, in the limit of large numbers, and especially in the right tail of the distribution, can be regarded as a Gaussian.

By differentiating the log-likelihood function with respect to ***σ*** and *p*^2^ and equating to zero, we find the maximum likelihood for the value of σ* that minimizes

${\sum_{i=1}^{n}\left(S_{i}-S(\sigma ,t_{i})\right)}^{2}$ – the minimum of the mean square error (MSE). In addition, this procedure provides the value of *p*^2^, which satisfies *p*^2^ = *MSE*(σ*).

The uncertainty in *σ*_1_ and *σ*_2_ is estimated by fitting for each *σ* to a Gaussian:

$$L(\sigma_{i})=C\;\exp\left[-\frac{n}{2MSE(\sigma^{*})}MSE(\sigma_{i})\right],$$

with the parameter’s uncertainty as its width, and *C* being a constant.

### Estimation results

**Fig 5:**
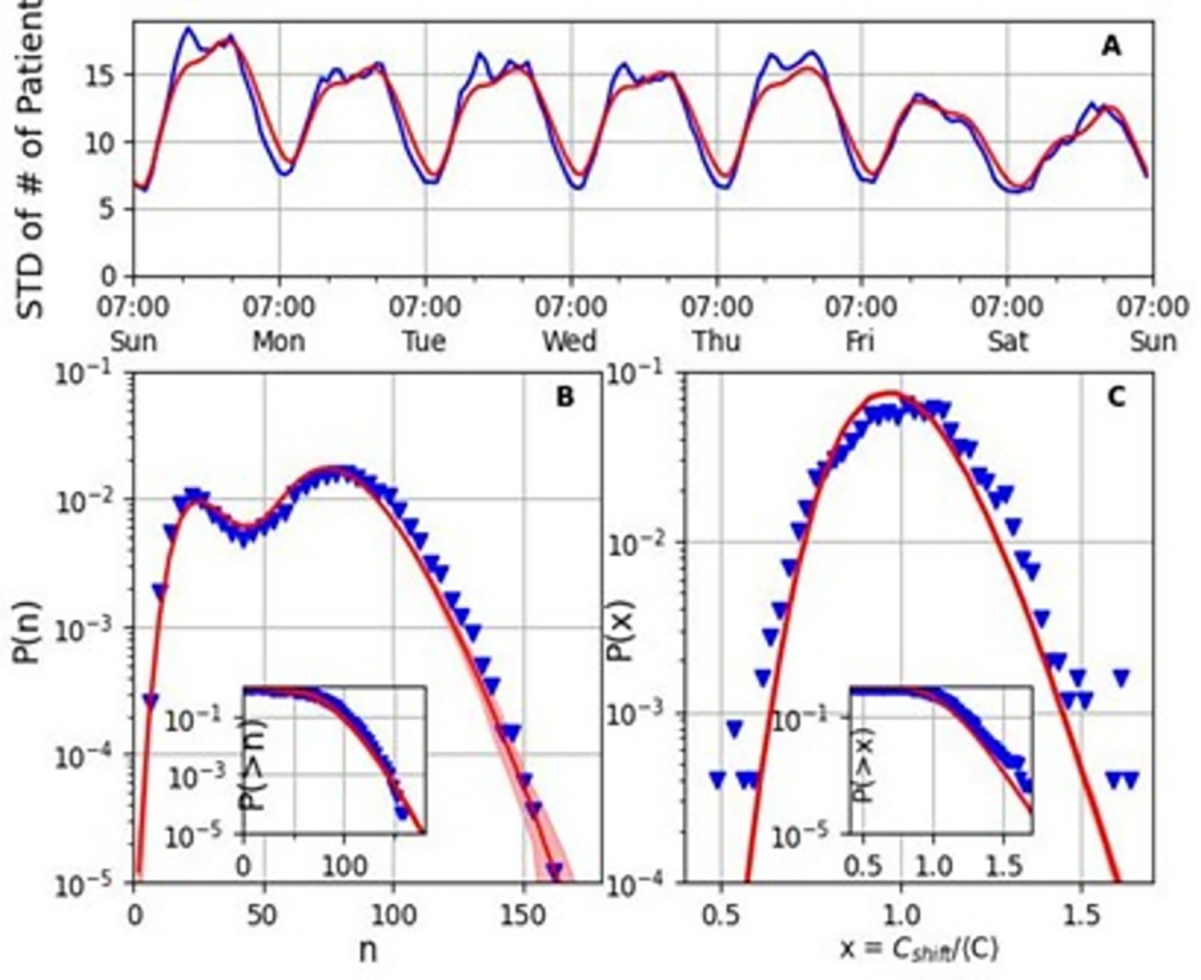
Model (red) fit with crowding statistics data (blue). (A) The fit of the hourly standard deviation of the number of patients. (B) Semi–logarithmic histogram of the number of patients in the ED in the 10 most crowded weekly shifts (Sunday through Thursday morning and afternoon). The shaded region accounts for the uncertainty in the estimation of the theoretical parameters. The inset shows the cumulative distribution. (C) Semi–logarithmic histogram of the patient hours of a shift relative to the average *x* = *C*_*shift*_/〈*C*〉 in the 10 most crowded weekly shifts. The inset shows the cumulative distribution. The *R*^2^ value for (A) is 0.921. The goodness–of–fit for (B) and (C), measured by the Kullback–Leibler divergence, is 0.02 and 0.05 respectively.

The estimated model enables prediction of the statistics of crowding events in the ED. **Fig 5**A shows excellent agreement between the model and data with respect to the standard deviation of the number of patients over the week. Furthermore, **Fig 5**B demonstrates that the prediction of the model for the entire patient-number distribution over a given ED shift agrees well with the distribution in the data (the inset shows the cumulative distribution).

Very good agreement with the data is also obtained when computing the distribution function for *relative* crowding, *x* = *C*_*shift*_/〈*C*〉, measured in the patient hours during a shift relative to the shift average (**Fig 5**C). For example, the probability to observe relative crowding between 1 and 1.1 (between average and 10% over average) is 24.4%±0.1% in the model compared to 24.0% of the data.

**Table 1** presents the values of the estimated parameters. Interestingly, although we did not fix the noise magnitude *σ*^1^ to the value of 1, to allow for additional sources of noise due to population heterogeneity, the maximum likelihood method estimated *σ*_1_≃1.1. This value is consistent with the approximation to the master equation by using the Langevin equation, for which *σ*_1_ = 1 (see SI, Appendix A in S1 File) [30]. We confirmed that fixing *σ*_1_ = 1 has a negligible effect on the results; the Kullback-Leibler divergence between the data and model patient-number distribution, as displayed and calculated in **Fig 5**B, changes from 0.02 to 0.029 in this case.

### Estimating overcrowding events

The ability to accurately predict the probability of overcrowding is an important tool for anticipating and exploring mitigation strategies for ED breakdowns. Overcrowding can occur when (i) the number of patients in the ED exceeds a certain absolute threshold, or (ii) when a large relative deviation above the average crowding occurs at a given time. While the absolute numbers indicate the load relative to the existing infrastructures (e.g., beds, physical capacity, staff availability), the relative definition is indicative of the subjective perception of crowding. 20 patients arriving unexpectedly to the ED can cause overcrowding if arriving over the weekend, but can be easily processed during weekdays, when the ED is usually prepared for many arrivals.

We follow the classification of NEDOCS [61], which ranks crowding levels on a scale from 1 to 6, where the top two levels are severe and dangerous overcrowding, which are respectively equivalent in our data to ~120 and ~140 patients. For example, the model’s prediction of the probability of observing dangerous overcrowding (>140 patients) is 0.085%±0.009%, compared to 0.077% in the data, see inset of **Fig 5**B. These probabilities are equivalent to ~7 hours per year. Remarkably, the accuracy of predicting such a rare event, occurring with probability <10^−3^, is within 10%.

In addition, our model provides excellent predictions for relative overcrowding. For example, the ratio of the probabilities of observing relative overcrowding greater than 40% and 20% is 0.086 in the data, and 0.074±0.01 in the model, within 15% accuracy (see inset of **Fig 5**C).

## Parameter elasticity of crowding

**Fig 6:**
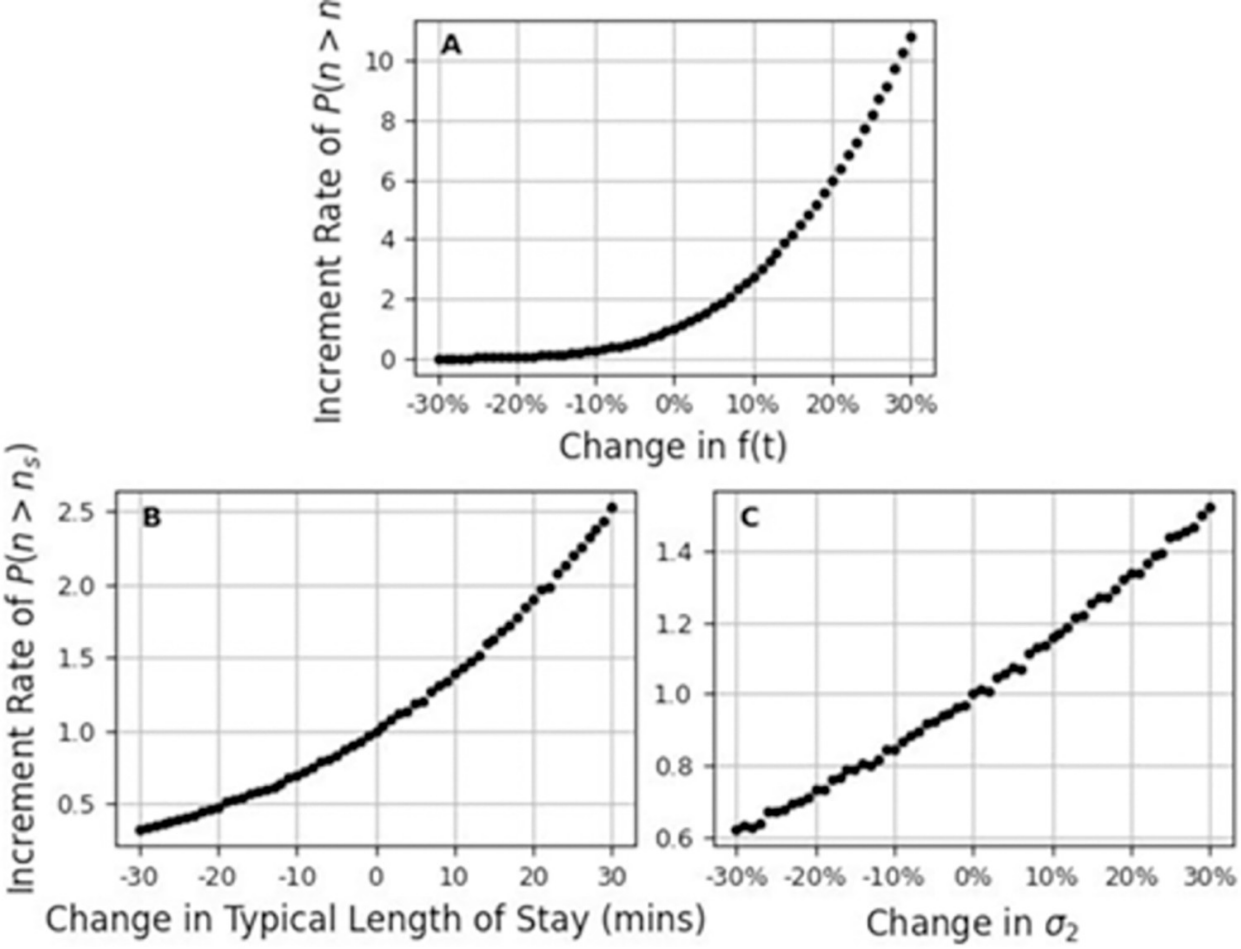
Parameter elasticity. The increment rate of the probability for severe or dangerous overcrowding: the ratio of P(n>n_s_) to P(n>n_s_) at zero change, as a function of the change in the (A) arrival flux f(t), (B) typical length of stay and (C) systematic noise magnitude σ_2_. Here n_s_ = 120 denotes the onset of severe overcrowding. Also, the average length of stay of the data is 3.9 hours, so approximately (depending on the weekday) a change in 10 minutes in the length of stay corresponds to 4%.

An important contribution of our model lies in its ability to explore the elasticity of crowding to arrival flux and length of stay. A change in the arrival flux *f*(*t*) can potentially occur due to population growth or changes in the medical condition of the population in the surrounding area. While the mean-field description [Eq (2)] indicates that the average number of patients, and the average patient hours depend linearly on the arrival flux *f*(*t*), surprisingly, the probability of observing severe or dangerous overcrowding, *P*(*n*>120), is highly non-linear in *f*(*t*). As shown in **Fig 6**A, while a 10% increment in the arrival flux increases the average number of patients by only 10%, the number of these extreme overcrowding events will increase by a factor of 2.8.

A patient’s average length of stay in the ED is given by 1/*β*, and is a metric that could potentially be mitigated by the ED management through better allocation of staff, or more efficient organizational procedures. **Fig 6B** and 6C show the change in the probability of overcrowding as a function of the change in 1/*β* (in minutes), and as a function of the change in *σ*_2_ (the amplitude of systematic noise), respectively. We find that overcrowding in the ED is extremely sensitive to the length of stay; e.g., shortening the typical length of stay by just 20 minutes (7–10% of the typical time, depending upon the day) reduces the number of severe and dangerous overcrowding events by 52% (**Fig 6**B). In addition, we find that the sensitivity to the length of stay is much higher than the sensitivity to systematic noise; e.g., lowering the systematic noise amplitude by 15% decreases the probability of a severe or dangerous overcrowding event by 22% (**Fig 6**C), whereas a similar decrease in this probability can be acquired by lowering the typical length of stay by just 2.5–3% (7 minutes).

Such calculations enable the ED to better allocate investments in various crowding mitigation initiatives. Reducing the typical length of stay (e.g., by addressing process bottlenecks, changing staff allocation, speeding up test results) is, as per our results, more effective in mitigating severe or dangerous overcrowding than reducing the systematic noise (e.g., by investing in maintenance and service contacts for equipment, avoiding fluctuations due to equipment down-time, better allocating critical equipment such as MRI scanners, or by cross functional training of staff to compensate for staff shortage).

## Limitations

This research has several limitations: First, being a stochastic model, this approach cannot predict a specific date of an overcrowding event, but rather, it provides the distribution and probability for such events to occur. Second, this study does not account for several low-frequency phenomena that can be found in the data, such as growth in demand over the years, or annual seasonality and systematic failures which may last several hours or days. Third, the model does not incorporate exogenous catastrophes such as large-scale accidents, extreme weather, or medical staff strikes. Finally, the model does not explicitly incorporate the medical staff size and composition, which may strongly affect the patients’ exit rate. Notably, these factors listed here are not expected to qualitatively change our results, especially those relating to the elasticity to various parameters, and the relative probability to have severe overcrowding. However, incorporating these additional elements into the model may improve the agreement with data with respect to absolute quantities, and more importantly, may provide additional means to mitigate ED overcrowding.

## Conclusion

This paper addresses crowded human environments, which are characterized by high volatility, variation across hours and days, overcrowding events, inter-individual heterogeneity and systematic noise. Modeling crowding in such a way as to account for all of these factors is critical for mitigating overcrowding and preventing service breakdowns.

We present a theoretical framework using stochastic population modeling, an approach that has been applied thus far to describe a broad range of natural phenomena, but not on crowding problems. The model captures the arrival flux, the exit rate, and includes a combination of additive and multiplicative noise. We implemented the model on data from a hospital emergency department, and found that our model provides adequate prediction of the momentary number of patients, the standard deviation and the patient number distribution. Notably, the model accurately predicts the probability of exceeding a certain crowding threshold. More importantly, the model’s main strength lies in its ability to predict how such overcrowding probabilities vary if the model parameters are changed. This ability, absent from various “black-box" models, is an important tool for the mitigation of overcrowding in the ED and the prevention of ED breakdowns.

We used the model to explore several important "what-if" questions: how does population growth affect severe overcrowding in the ED? What happens if the length of stay is shortened by a certain amount? And: What is the effect of changing the systematic noise.

This work is important to both practice and research. ED practitioners can use the model to better allocate resources as per their expected effect. They can predict the volatility and rate of expected overcrowding events of any given magnitude. On the theoretical side, this work demonstrates the power of the stochastic population dynamics formalism, which has been widely used for describing various effects, e.g., in population biology, ecology, epidemiology, chemistry, and statistical physics. We show that this formalism can also be applied to describe the dynamics of populous human environments. The latter, while influenced by numerous variables and characterized by high volatility and overcrowding spikes, can be captured by a compact analytic formulation that enables revealing key underlying mechanisms, and obtaining important insights as to the role of various governing parameters.

This work paves the way for future research in several directions. First, the model can be extended to include the medical staff size as an additional dynamic variable, which can allow computing elasticity to parameters such as the mean staff size and its typical fluctuations. Furthermore, one can study the determinants of other model parameters, such as the dependence of exit rates on the total population in the ED, staff fatigue, medical team composition, etc. Another avenue is to complement the model with other stochastic behaviors, such as low-frequency trends and noises, or an arrival flux with stochastic burst generators mimicking large accidents or other catastrophes. Finally, the model, with these possible generalizations, can be implemented on other populous environments (public transportation, shopping malls, etc.).

## Methods

### Ethics

We used the records as appeared in the ED database (after concealing the patients’ identities). The patients’ identifiers were encrypted by the hospital prior to delivery, so the research team had no access to any information that could identify individual patients during or after data collection. All records relate to visits prior to the beginning of the research. The research project started effectively about 8 months after the date of the last medical record (the data were accessed between Feb 28^th^, 2019 and Feb 23^rd^, 2021). Therefore, the patients’ treatment at the ED was not affected by the research. No external intervention was made, no experiments were done, and no questionnaires were distributed. Since this is a retrospective study of medical records and data were analyzed anonymously, no informed consent is required.

The data collection and study were approved by the institutional ERB at Shamir Medical Center, request no. 0028-29-ASF. In writing this paper we followed as closely as possible the STROBE reporting conventions of observational human subject research.

## Supporting information

S1 File Contains the A); Governing Equations for the Stochastic Model. B) Accounting for Colored Noise; C) Alternative Models for the Arrival Flux.
(DOCX)
