# Correction: Estimating emergency department crowding with stochastic population models

**DOI:** 10.1371/journal.pone.0304152

**Published:** 2024-05-16

**Authors:** The PLOS ONE Staff

## Notice of republication

This article was republished on May 1, 2024, to correct errors in equations that were introduced during the typesetting process. The publisher apologizes for the errors. Please download the article again to view the corrected version. Both the originally published, uncorrected article and the republished, corrected version are provided here for reference.

## Supporting information

S1 File Originally published, uncorrected article. (PDF)

S2 File Republished, corrected article. (PDF)
