# Supplementary material for: Estimating emergency department crowding with stochastic population models
Source: PLoS One. 2023 Dec 1;18(12):e0295130. doi: 10.1371/journal.pone.0295130 (PMC10691698; doi:10.1371/journal.pone.0295130)
Supplement: S1 File — Contains the A); Governing Equations for the Stochastic Model. B) Accounting for Colored Noise; C) Alternative Models for the Arrival Flux. (DOCX) [file pone.0295130.s001.docx]

**Supporting Information**

Gil Parnass^1^, Osnat Levtzion-Korach^2^, Renana Peres^3†^, Michael Assaf^1†^,

1 Racah Institute of Physics, Hebrew University of Jerusalem, Jerusalem 91904, Israel

2 Shamir Medical Center, Be’er Ya’akov, Israel

3 The Hebrew University Business school, Jerusalem, 91905, Israel

† To whom correspondence should be addressed * renana.peres@mail.huji.ac.il,

michael.assaf@mail.huji.ac.il

1. **Appendix A: Governing Equations for the Stochastic Model**

**A.1 Influx-Outflux Process**

The stochastic dynamics of incoming and outgoing patients consist of two stochastic processes: creation ("influx" or "arrival") and decay ("outflux" or "exit") of particles, which can be written as

$$\emptyset\underset{\to}{f} A, A\underset{\to}{\beta}\emptyset.$$

(S1)

Note that the rate $f$ is absolute, and $\beta$ is per particle. The dynamics described by this set of reactions is often used to model various chemical processes, or more generally, any Poisson process.

**A.2 Deterministic Rate Equation**

At the deterministic level, the average number of particles$\bar{n}$ as a function of time satisfies the following rate equation:

$$\dot{\bar{n}}= f -\beta\bar{n}\left( t \right).$$

(S2)

This equation is obtained in a straightforward manner from rates (S1). The population growth rate is given by$f$, while the degradation rate per individual is given by$\beta$. The dynamics of Eq.(S2) are simple, and can be solved analytically. The equation admits an attracting fixed point at$\bar{n}_{*}= f/\beta$. Upon starting at any given$n_{0} > 0$, the system will converge to the fixed point after a typical timescale on the order of$\tau=\beta^{-1}$. The exact solution reads:

$$\bar{n}(t) = \bar{n}_{*}+\left( n_{0}-\bar{n}_{*} \right)e^{-\beta t}.$$

(S3)

Note that, the rate equation (here, and in general) describes the average behaviour of the system, and ignores demographic fluctuations. This is justified as long as the typical population size satisfies$N\gg1$. Notably, Eqs.(S2) and (S3) are the time-independent version of Eqs.(1) and (2) in the main text, respectively. The numerical solution of the rate equation for our case can be seen in Fig. 4.

To account for the intrinsic noise related to the discreteness of particles and stochasticity of the particles involved, a master equation can be used.

**A.3 Stochastic Case: the Master Equation**

The master equation is a gain-loss equation, describing the evolution of the probability$P_{n}(t)$ of observing n particles at a given time t, where n is discrete and t is taken to be continuous. For the stochastic process described by rates (S1), the master equation yields:

$$\frac{dP_{n}\left( t \right)}{dt} = f[P_{n-1}(t) - P_{n}(t)] +\beta[\left( n+1 \right)P_{n+1}(t) - nP_{n}(t)].$$

(S4)

The solution of this equation,$\left\{ P_{n}\left( t \right) \right\}_{n=0}^{\infty}$, for any n and t, yields the probability distribution function (PDF) of the system, which describes typical fluctuations, as well as rare events of interest. When the rates$f$ and$\beta$ are explicitly time dependent, an analytical solution of Eq.(S4) is unknown in general. However, in the time-independent case, a solution can be found, e.g., using the method of characteristics [1].

Here we provide the stationary solution for the PDF of Eq.(S4). Letting$\dot{P}_{n}(t) = 0$ enables finding a recursive relationship between$P_{n}$ and$P_{n-1}$. After some algebra, we obtain:

$$P_{n}=P_{0} \prod_{k=0}^{n-1} \frac{f}{\beta(k+1)}= P_{0}\left( \frac{f}{\beta} \right)^{n}\frac{1}{n!} ,$$

(S5)

Where$P_{0}$ is found from the normalization condition: $\sum_{n=0}^{\infty} P_{n} = 1$. This PDF is simply a Poisson distribution with mean$\lambda= f/\beta$, and therefore, one finds$P_{0}=e^{-\lambda}= e^{-f/\beta}$.

Notably, the rate equation can be directly obtained from the master equation (S4). Multiplying Eq.(S4) by n, summing over all$n$'s, and using the relation$\bar{n}=\sum_{n} nP_{n}$, we obtain Eq.(S2) upon neglecting subleading-order terms and assuming$N\gg1$.

**A.4 The Fokker-Planck and Langevin Equations for the Influx-Outflux Process**

The Fokker-Planck equation (FPE) or Langevin equation are best known from applications of classical mechanics, and describe the dynamics of a particle moving in a deterministic force field with some noise.

When the starting point is a master equation, the FPE can be obtained using the van-Kampen system size expansion [1] [2], valid for large$n$. In the case of the influx-outflux process (S1), the FPE yields:

$$\frac{\partial P\left( n,t \right)}{\partial t}=-\frac{\partial}{\partial n}\left[ P\left( n,t \right)\left( f-\beta n \right) \right]+\frac{1}{2}\frac{\partial^{2}}{\partial n^{2}}\left[ P\left( n,t \right)\left( f+\beta n \right) \right].$$

(S6)

In the time-independent case, the stationary PDF is found by equating the left-hand side of Eq.(S6) to zero, and using the zero-flux boundary condition:

$$P\left( n \right)=C\exp\left( 2\int_{0}^{n} \frac{f-\beta n^{'}}{f + \beta n^{'}}dn^{'} \right),$$

(S7)

where C is a normalization factor, found by demanding that the PDF be normalized to 1. To test the quality of the Fokker-Planck approximation, we compare in ***Fig. S1*** the solution of the master equation to that of the FPE. As expected, as the typical value of$n$ increases, the accuracy of the FPE improves.


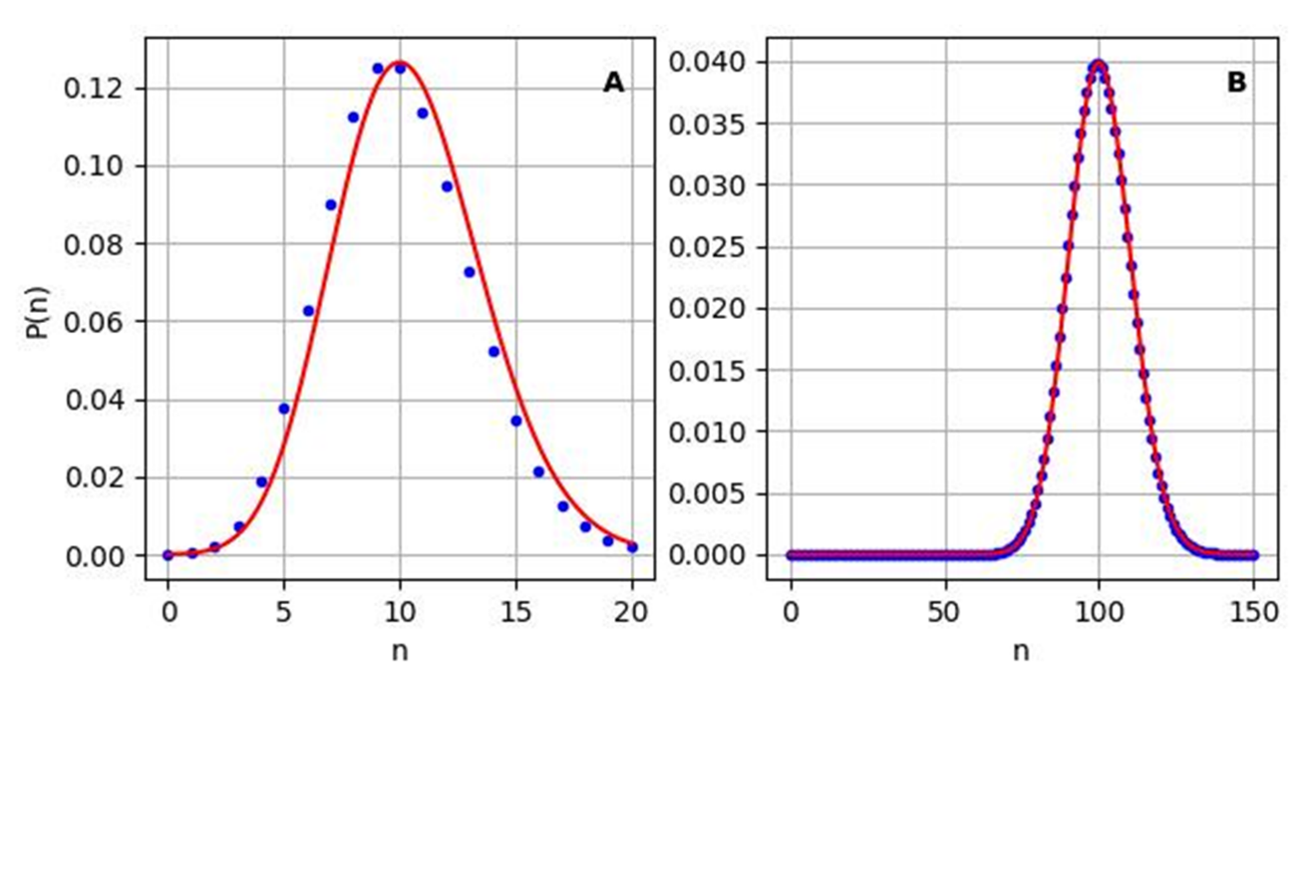


***Fig. S1*** Comparison between the solution of the master equation [Eq.(S5)] (blue dots) and that of the Fokker Planck Equation [Eq.(S7)] (red line) for 2 different cases

(A) $f=5, \beta=0.5, n_{*} = f/\beta= 10,$ (B)$f=25, \beta=0.25, n_{*} = f/\beta=100.$

Notably, the equivalent Langevin equation to the FPE, given by Eq.(S6), reads:

$$\frac{dn\left( t \right)}{dt} = f -\beta n(t) +\sqrt{f+\beta n\left( t \right)} \eta(t).$$

(S8)

This equation describes the randomly-varying number of patients subject to deterministic forcing,$f-\beta n$, and multiplicative noise,$\sqrt{f+\beta n\left( t \right)} \eta(t)$, where$\eta(t)$ is delta-correlated Gauassian noise.

Equation (S8) is similar to Langevin equation in the main text [Eq. (3)], with time-independent rates,$\sigma_{1}=1$, and without systematic noise ($\sigma_{2} =0$). Note that, in the case of explicitly time-dependent rates, in general the PDF cannot be found analytically, and one has to resort to numerical techniques.

1. **Appendix B: Accounting for Colored Noise**

Here we consider the scenario where both the internal and external noise have a finite correlation time. Consequently, instead of taking delta-correlated noise, we complement the Langevin equation for the fluctuating patients number [Eq. (3) in the main text] with two Ornstein-Uhlenbeck equations:

$$\frac{d\xi_{1}\left( t \right)}{dt}=\frac{\xi_{1}\left( t \right)}{\tau_{1}}+ \sqrt{\frac{2\sigma_{1}^{2}}{\tau_{1}}}\zeta_{1}\left( t \right),$$

$$\frac{d\xi_{2}\left( t \right)}{dt}=\frac{\xi_{2}\left( t \right)}{\tau_{2}}+ \sqrt{\frac{2\sigma_{2}^{2}}{\tau_{2}}}\zeta_{2}\left( t \right)$$

Where$\sigma_{i}$ are the amplitudes of the noise,$\tau_{i}$ are the noise correlation times, and$\zeta_{i}$ are delta-correlated Gaussian noise terms.

To study these scenarios, we used the same maximum likelihood estimation method (as described in Methods section), and fitted$\sigma_{1},\sigma_{2}$ for each pair of noise correlation times, see ***Table S1***. To do so, we calculated the Kullback-Leibler divergence, for the same case of the 10 most crowded shifts of the week (as done in Fig.5B), see ***Table S1***. The results do not vary much from the value of$0.02$ that we obtained for the delta-correlated case (which corresponds to the case of$\tau_{1},\tau_{2} = 1$, the shortest correlation time possible for this dataset), and range from 0.016 to 0.032. This implies that while our choice of delta-correlated noise was arbitrary, accounting for finite correlation time of the noise does not introduce notable changes to the model's predictions.

**Table S1.** Kullback–Leibler (KL) divergence between the data and model for the patient-number distribution of the 10 most busy shifts, for correlated noise (Uhlenbeck-Ornstein noise) with various correlation times

1. **Alternative Models for the Arrival Flux**

We implemented several other population dynamics models based on the Langevin equation, using several other arrival fluxes. For example, we implemented the model described by Eq. (3) using a simple trapezoid function of the form:

$$f\left( t \right)=\left\{ \begin{matrix} a_{1}^{i}+\frac{a_{2}^{i}-a_{1}^{i}}{t_{2}-t_{1}}(t-t_{1}) & t_{1} < t < t_{2} \\ b & else \end{matrix} , \right.$$

(S9)

Where$a_{1}^{i},a_{2}^{i}>b$ are the rates at the rush hours, with$i$ (here and below) indicating various parts of the week ($i\in\{Sunday, Midweek, Friday, Saturday\}$); and$b$ is the arrival flux at the off-peak hours (mostly during the night or early morning).$\beta(t)$ differs between the various parts of the week, such that$\beta$ is taken to be$\beta^{i}$.

Notably, we also tested a simpler, rectangular arrival flux, which has a constant value during rush hours:$a_{1}^{i} = a_{2}^{i}$ for every part of the week. The fitted arrival flux functions can be seen in ***Fig. S2***A. For each of the models we repeated the process of fitting the stochastic components. The fitted parameters can be seen in **Table S2**.

Using the various models we estimated the patient number distributions for every model. ***Figures S2***B and ***S2***C show excellent agreement between the crowding statistics of the models and data. This indicates that using simplified approximate functional forms of the arrival flux allows for a semi-analytical calculation of the PDF, as well as parameter elasticities, as done numerically in the main text.

Finally, ***Fig. S3*** illustrates the relative fluctuations in the total number of patients with respect to the hourly average,$(n(t)-\langle n\left( t \right)\rangle)/\langle n\left( t \right)\rangle$ over a typical week, for the data and the various models (see also Fig. 1 in the main text). The figure shows that the relative fluctuations over the week can be very high, reaching 75% above and 60% below the average number of patients over just a few days. One can also see that it takes several hours for the ED to revert to the average crowding levels. This stresses the importance of accounting for noise while modeling crowding in such environments.


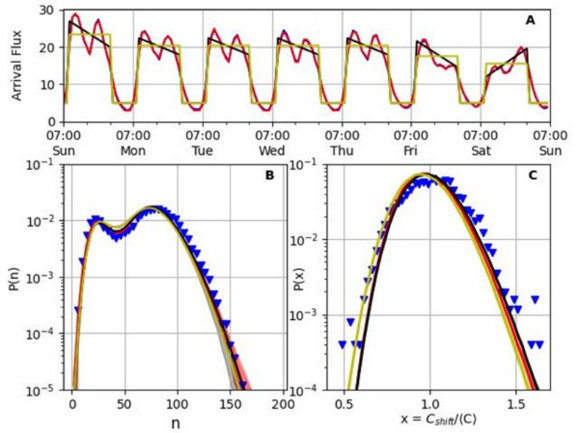


***Fig. S2*** Crowding statistics of the data (blue triangles) fit with three arrival flux models (measured arrival flux in red, trapezoid in black, rectangular in yellow). (A) The fit of the hourly arrival flux. (B) Semi-logarithmic histogram of the number of patients in the ED in the 10 most crowded weekly shifts (Sunday through Thursday morning and afternoon). The shaded region accounts for the uncertainty in the estimation of the theoretical parameters. *(C) Semi-logarithmic histogram of the patient hours of a shift relative to the average* $x= C_{shift}/\langle C\rangle$ *in the 10 most crowded weekly shifts.*

**Table S2** Fitted parameter values, for the measured arrival flux (A), trapezoid arrival-flux model (B), rectangular arrival-flux model (C). Here model (C) is similar to model (B), see Eq. (S9), but with a constant value during rush hours:$a_{1}^{i}=a_{2}^{i}$ for every part of the week. Model (C) is the one used in the main text.


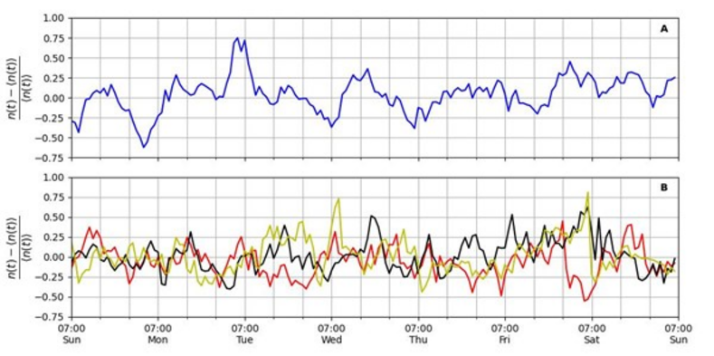


***Fig. S3*** Qualitative comparison of the data and model. (A) The normalized deviation in the total number of patients relative to the average,$(n(t)-\langle n\left( t \right)\rangle)/\langle n\left( t \right)\rangle,$ in a typical week in the data.

(B) The normalized deviation in the total number of patients relative to the average in a typical realization of a week by our simulation using the various arrival flux models (measured in red, trapezoid in black, rectangular in yellow).

# References

| [1] | C. W. Gardiner, Handbook of stochastic methods for physics, chemistry and the natural sciences, Second ed., vol. 13, H. Haken, Ed., Berlin: Springer-Verlag, 1985. |
| --- | --- |
| [2] | M. Assaf and B. Meerson, "WKB theory of large deviations in stochastic populations," *Journal of Physics A: Mathematical and Theoretical,* vol. 50, p. 263001, 2017. |
